# Healthy people and healthy profits? Elaborating a conceptual framework for governing the commercial determinants of non-communicable diseases and identifying options for reducing risk exposure

**DOI:** 10.1186/s12992-017-0255-3

**Published:** 2017-06-15

**Authors:** Kent Buse, Sonja Tanaka, Sarah Hawkes

**Affiliations:** 1 UNAIDS, Geneva, Switzerland; 2 Paris, France; 3 Institute for Global Health, University College London, London, UK

**Keywords:** NCD governance, Commercial drivers, Public-private regulation, Self-regulation, Partnership

## Abstract

**Background:**

Non-communicable diseases (NCDs) represent a significant threat to human health and well-being, and carry significant implications for economic development and health care and other costs for governments and business, families and individuals. Risks for many of the major NCDs are associated with the production, marketing and consumption of commercially produced food and drink, particularly those containing sugar, salt and transfats (in ultra-processed products), alcohol and tobacco. The problems inherent in primary prevention of NCDs have received relatively little attention from international organizations, national governments and civil society, especially when compared to the attention paid to secondary and tertiary prevention regimes (i.e. those focused on provision of medical treatment and long-term clinical management). This may in part reflect that until recently the NCDs have not been deemed a priority on the overall global health agenda. Low political priority may also be due in part to the complexity inherent in implementing feasible and acceptable interventions, such as increased taxation or regulation of access, particularly given the need to coordinate action beyond the health sector. More fundamentally, governing determinants of risk frequently brings public health into conflict with the interests of profit-driven food, beverage, alcohol and tobacco industries.

**Materials:**

We use a conceptual framework to review three models of governance of NCD risk: self-regulation by industry; hybrid models of public-private engagement; and public sector regulation. We analyse the challenges inherent in each model, and review what is known (or not) about their impact on NCD outcomes.

**Conclusion:**

While piecemeal efforts have been established, we argue that mechanisms to control the commercial determinants of NCDs are inadequate and efforts at remedial action too limited. Our paper sets out an agenda to strengthen each of the three governance models. We identify reforms that will be needed to the global health architecture to govern NCD risks, including to strengthen its ability to consolidate the collective power of diverse stakeholders, its authority to develop and enforce clear measures to address risks, as well as establish monitoring and rights-based accountability systems across all actors to drive measurable, equitable and sustainable progress in reducing the global burden of NCDs.

## Background

The incoming Director-General of the World Health Organization will face many challenges, and non-communicable diseases (NCDs) have been identified as among the top priorities [1]. Growing burdens of NCDs and rising rates of risk exposure, particularly in low- and middle-income countries (LMICs), are a major impediment to the 2030 Agenda for Sustainable Development (Agenda 2030) and the Sustainable Development Goals (SDGs). NCDs – notably cardiovascular diseases, cancers, chronic respiratory diseases and diabetes – have become the leading cause of premature death and disability [2], including exerting a high impact on the health of the poorest billion [3].

While recognizing that there are a range of determinants of NCDs, in this paper we are concerned with the risks inherent from consumption of, or exposure to, commercial products – such as ultra-processed foods and beverages, tobacco and alcohol. The role of the so-called “commercial determinants of health” [4] has been described in both the health governance and health promotion literature over the past few years, as driving risks of ill-health [5–10].

In this paper we argue that grappling with the commercial drivers of ill-health requires addressing the “profit-driven epidemics” [11] that characterize some NCDs. We do not attempt to quantify the exact extent to which commercial drivers can be held directly responsible for specific NCD-related health outcomes (the “population attributable fraction”) but believe it is likely to be substantial, and rising particularly in low- and middle-income countries (LMIC) where virtually all “Big Food” and “Big Tobacco” sales growth is occurring [12, 13]. The rapid penetration of transnational food and drink companies into emerging global markets [14] and increased consumption of ultra-processed foods/drinks – containing added sugars, high levels of salt, and transfats – tracks closely with rising levels of child and adult obesity, diabetes and cardiovascular diseases [15]. The 2011 report of the UN Special Rapporteur on the Right to Health submitted to the UN General Assembly recognized the transnational corporations of the global food industry as “the primary driver of diet-related NCDs” [16].

The international community has begun to acknowledge the scale of the crisis and its responsibility in curbing the NCD epidemic. In 2011, the United Nations General Assembly convened its first High-Level Meeting on NCDs [17], and a second in 2014 [18], both of which urged Member States to adopt time-bound commitments. More recently, the adoption of Agenda 2030 significantly expanded the range of global development priorities, and target 3.4 specifically calls for a reduction by “one third premature mortality from NCDs through prevention and treatment” [19]. Nonetheless, it is worth noting that in 2015 < 1% of all financial development assistance for health was targeted to NCDs [20].

The slow response of the global public health community to the NCD crisis may be due to a range of factors. Firstly, there is an inherent institutional path dependency in global health institutions that impedes changing ineffective governance processes, priorities and policies [21]. Secondly, there has been tepid civil society activism demanding political priority and action – despite the previous successes of civil society in major areas of global health, such as AIDS [22] or other areas of pro-poor policy reform [23]. While some civil society organisations and alliances are tackling NCDs – e.g. the NCD Alliance and NCDfree - these institutions have yet to achieve the level of cohesion, activism or political acumen that has characterised other public health accomplishments. Indeed, the NCD community (including civil society) was criticised as recently as “semi-comatose” by the editor of a leading medical journal [24].

Thirdly, as the current Director General of WHO has noted “Efforts to prevent noncommunicable diseases go against the business interests of powerful economic operators” [25]. The profits at stake are vast, particularly when compared to the available public finances to implement health-protecting and promoting policies. The combined market capitalisation of the five largest tobacco corporations is more than US$400 billion and $600 billion for the five largest beverage firms [26], the latter exceeding the GDP of all but the world’s 20 wealthiest countries. Annual profits in the tobacco industry exceed US$35 billion [27], and in the USA alone, the tobacco industry spent US$8.9 billion on marketing in 2013 [28]. In contrast, the annual budget of WHO is approximately US$2.2 billion [29].

We cannot treat our way out of the NCD epidemic. Universal access to NCD treatment, will bear a prohibitive price tag for any country, even the wealthiest. In the United States, for example, the economic burden of treating diabetes has skyrocketed: in 2012 alone, medical costs to treat the 30 million Americans living with the disease reached $176 billion [30]. In China, the annual cost of diabetes care exceeds the total health expenditure per capita in the country [31]. Treating China’s 114 million people estimated to have diabetes would cost the country some $48 billion annually [32]. Premature NCD-related deaths in LMICs will result in estimated economic losses of US$7 trillion over the next 15 years and trap millions of people in poverty [33]. The value of life lost, including lost income, out-of-pocket spending related to medical care, and pain and suffering due to NCDs, is estimated to double between 2010 and 2030 [34].

Effective prevention solutions are urgently needed. Cost-effective policy/regulatory interventions such as tax and price increases on tobacco products or alcohol [35] or regulating food/beverage marketing aimed at children [36] could prevent a large proportion of NCD-associated illness and death [37], but risk putting the health community in conflict with powerful stakeholders [1, 38, 39]. The health sector has had some success in reducing NCD risk exposure. For example, the Framework Convention on Tobacco Control (FCTC) and its implementation show that it is possible to challenge industry interests and improve global health [40]. The global health community, led by the World Health Organization (WHO), has been unequivocal in its refusal to collaborate with the tobacco industry [41]. Yet although there are valuable lessons from the FCTC, implementation remains voluntary and, hence, uneven [42]. Furthermore, the immediate use of similar governance mechanisms in other sectors (e.g. alcohol) has been called into question owing to likely low levels of political feasibility, among other considerations [43].

While the need for a global collective response and policy coherence across sectors to effectively hold the commercial food, beverage, alcohol and tobacco industries (at least partially) accountable for public health outcomes is clear, the question of ‘how’ requires further consideration. In this paper, we pose three questions: Is the current governance architecture for global health fit to manage the governance of the commercial determinants of ill-health? What models for governance of commercial determinants currently exist, and how well are they working? And, given the influence of private authority on agenda-setting and policy formulation, what is the scope for enhancing transboundary legal and prescriptive frameworks to protect health?

### Conceptual framework and methods

We applied a conceptual framework proposed by Buse and Naylor [44], for classifying the involvement of the commercial sector in global governance for health. The framework presents three models of interaction between public and private sectors – the relationship that sits at the core of how we achieve goals of improving population health through reduction of exposure to the commercial determinants of health. The three models are: self-regulation by industry; regulation through partnership; and regulation of the private sector by the public sector.

We reviewed the health governance literature looking for examples of regulation and/or co-operation with private industry in order to achieve population health gains. Given the extensive literature on tobacco control, we focused our efforts mainly on reviewing other commercial determinants – namely ultra-processed foods and beverages, and the alcohol industry – but drew from the tobacco literature where necessary. Our focus was on models of governance, and particularly where these models had been evaluated. We used keywords (noncommunicable diseases, sugar, salt, sodium, transfats, alcohol, tobacco, combined with governance, commercial determinants, industry, private or public) and our review was limited to papers published in English available in selected databases (JSTOR, PubMed, SCOPUS). While not definitive, the review provides examples within each of the Buse/Naylor models of interaction, and we use these to explore potential forms of effective governance in reaching population health goals in controlling the commercial determinants of NCDs.

## Findings

### Industry self-regulation

Voluntary actions by business are popular with the commercial sector and serve several functions including: responding to concerns of consumers thereby increasing sales or staving off boycotts; differentiating a firm from competitors that do not participate; and preventing or delaying statutory regulation – indeed self-regulation is often implemented in response to a threat of public regulation [33]. From a political perspective, voluntary actions can, at times, have benefits over statutory regulation – they involve relatively low levels of public expenditure and may generate better compliance than intergovernmental and national regulation where enforcement may be less effective and more costly.

‘Big Food’, in particular, is keen to demonstrate that it is actively responding to NCD epidemics. This has most visibly taken the form of voluntary codes of conduct and highly publicized pledges. The International Food and Beverage Alliance (IFBA), for example, is a group of 11 major companies including Coca-Cola, Mondelēz, Nestlé and Unilever, formed in 2008 with a focus on tackling NCDs through a range of initiatives such as product reformulation to reduce sodium and sugar, and promote responsible marketing to children [45]. However, an evaluation of the self-regulatory Children’s Food and Beverage Advertising Initiative (CAI) implemented by 17 food and beverage manufacturers in Canada, found that despite the pledge to devote 50% of their child-focused multi-media advertising to “healthier dietary choices” and (for 8 companies) not to direct any advertising to children under 12 years, CAI companies were responsible for more food promotions and advertised foods were higher in fats, sugar and sodium than a comparison from non-CAI companies. The authors of the evaluation concluded that some companies are “simply paying lip service to the childhood obesity crisis” and “governments must consider regulatory approaches in marketing to children” [46].

In the United Kingdom, voluntary initiatives to reduce salt intake to combat hypertension through product reformulation have contributed (along with consumer behavior change campaigns and enhanced product labelling) to some success in reducing overall levels of salt consumption [47] – from 2001 to 2011 average salt intake in the UK declined from 9.5 g/day to 8.1 g/day (9.3 g/day in men and 6.8 g/day in women), a figure still far higher than the UK’s Food Standards Agency goal of 6 g/day [48]. In its current iteration, the UK Public Health Responsibility Deal promotes voluntary agreements with industry to help achieve the national salt targets. Reeve and Magnusson in their comparison of salt reduction programmes in both the UK and the USA highlight a number of governance concerns with the concept of the (voluntary) Responsibility Deal, including: industry has a disproportionate level of influence on the Deal’s governing bodies; lack of specific and time-bound commitments for action; inadequate monitoring mechanisms; lack of participation by some sections of industry (including smaller independent food service establishments and caterers); and, perhaps most importantly, lack of any enforcement options [39].

Voluntary regulation by the tobacco industry has exposed longer-term concerns and potential negative outcomes associated with self-regulation. An analysis by Hiilamo and Glantz [49] of the use of health warning labels (HWL) on cigarette packets found that the move to FCTC-compliant labelling (which is more graphic, occupies a mandated proportion of the packet, and has been found to have some positive impact on tobacco control [50]) was more likely to occur in those countries that “avoided agreements with the tobacco industry in the early 1990s”.In other words, in those countries where the tobacco industry had voluntarily adopted early self-regulation of packaging there was a stronger likelihood that more stringent mandated packaging rules would not be implemented in the future. The authors concluded that there is a need for “vigilance against any industry efforts to circumvent mandatory policies through voluntary arrangements” and highlighted the experience of tobacco control as having lessons for alcohol and processed food.

Lessons from other sectors may include measures such as the “private regulation of global corporate conduct” [51] which is defined as permitting a role for non-governmental organisations (NGOs) to participate in the ‘civil regulation’ of global companies, e.g. the Marine or Forestry Stewardship Councils. Such an approach is often prompted by a company’s decision to respond to actual or threatened public criticism, or promoted by notions of corporate social responsibility to encourage concepts of “good global corporate citizenship” [45] and perceived as being ‘good for business’. These private regulations of the corporate sector have been reviewed as being less effective than regulation by the public sector, and probably of comparable effectiveness to simple self-regulation, in developed economies, but possibly more effective than regulation when the state is weak or fragile.

While clearly the preferred approach of industry, as well as the default approach of many governments and the UN, many observers remain doubtful of the capacity of self-regulation to effectively govern global health concerns, particularly in the absence of independent (public) accountability mechanisms [52] or other standards for effective self-regulation. The NCD Action Group of The Lancet, was skeptical of self-regulation, stating: “Despite common reliance on industry self-regulation [and public-private partnerships], there is no evidence of their effectiveness or safety” [26]. Reviews of self-regulation mechanisms across different industries have identified a number of standards that would need to be met for self-regulation to be effective. These include multi-stakeholder governance mechanisms with no single party having disproportionate power, external and objective evaluation mechanisms including mandatory public reporting of adherence to codes, and oversight by an appropriate regulatory or health body [53].

### Co-regulation through partnership

Engagement between public and private sectors in health has expanded rapidly in the past two decades – both through formal partnership mechanisms and through looser arrangements between the sectors [54]. SDG 17 has a target to “encourage and promote effective public, public-private and civil society partnerships”, and the UN system is promoting this concept at the highest levels. In December 2016 the UN General Assembly granted observer status to the International Chamber of Commerce – the world’s largest business organization with more than 6 million members – thus elevating the private sector to the status of having a direct voice at the highest level of the UN, since those with observer status are permitted to make interventions [55]. However, close ties between the UN system and the food and beverage industries, for example, have raised concerns of the potential for industry to wield influence over global health policy [56]. Previous experience urges future caution: through their participation in the Global Health Council’s NCD Roundtable, PepsiCo and Coca-Cola contributed to policy recommendations to the 2011 UN High-Level Meeting on NCDs (HLM) and sponsored side-events at the UN [57]. The Conflicts of Interest Coalition emerged from concerns about such influence at the HLM, and called on the UN to “protect public health policy from commercial interests” [58].

Several World Health Assembly resolutions, beginning in 2004, have called on WHO to engage with private sector actors [59], and all three WHO-backed global strategies on NCDs [60–62] advocate for partnership and cooperation with private actors. In May 2016 the World Health Assembly passed a resolution on the Framework of Engagement with Non-State Actors (FENSA) – which include “nongovernmental organizations [NGOs], private sector entities, philanthropic foundations and academic institutions” [63]. Industry welcomed FENSA as providing an opportunity for reaching shared goals “to make this world healthier” [64], but NGOs raised concerns over potential “problematic entanglements” [65]. While FENSA established rules of engagement between WHO and non-state actors, the framework has been criticised (by ourselves) as not containing sufficient guidance on governing the activities of industry in relation to public health outcomes [66].

WHO’s institutional commitment to preventing and managing conflicts of interest with industry is unambiguous, but the scope of the challenge in relation to commercial determinants of NCDs may be impossible to govern – particularly given the wide variety of private sector representatives who may be looking for a seat at the decision-making Table. A Reuters investigation found at least two of the 15 members of WHO’s Nutrition Guidance Expert Advisory Group had direct financial ties to the food industry [67]. In addition, WHO’s financial insecurity may be creating new space for the engagement of the food and beverage industry [26]. In 2012, WHO’s Latin American regional office, the Pan American Health Organization (PAHO), accepted hundreds of thousands of dollars of industry funding – including from Coca-Cola, Nestlé and Unilever (though such donations are prohibited for Geneva headquarters and the other regional offices) [68]. Some critics warn that any partnership creates benefit for industry (indeed industry *must* benefit for sustained engagement) – but see no clear, established or legitimate mechanism through which public health would be protected [1].

Multilateral institutions engage in at least two public-private partnerships which address NCDs. The Scaling Up Nutrition (SUN) Movement is a multisectoral partnership (whose Lead Group operates under the auspices of the UN Secretary General) established to ensure the right to food and nutrition, including a focus on SDG targets to halt the rise in child, adolescent and adult obesity rates and see no increase in diabetes. An independent evaluation of SUN conducted in 2015 noted “hostility” and “resistance” within the SUN movement to collaboration with the business sector, and concluded that SUN’s challenge was to turn “high quality printed and online media” into practical action [69]. The EAT Foundation established in 2016 [70], funded by private philanthropic foundations, has Strategic Advisers and Advisory Board members from the UN system (WHO, World Food Programme, among others) as well as from private-for-profit companies (such as Google) and aims to “develop practical guidelines for consumers and the private sector” and “to change consumer behavior at the population level”. Their work does not yet appear to have been evaluated.

At the national level, public-private partnerships have tended to focus on issues around food supply, food safety and access to treatment (including in low- and middle-income countries). The smaller number of examples of partnership to tackle NCD prevention directly have met with mixed success. US First Lady Michelle Obama centred her anti-obesity initiative, ‘Let’s Move’, around creating public–private partnerships that, for the first time, set national goals to ‘end childhood obesity in a generation’ [71]. Surpassing its initial calorie-reduction pledge towards advancing the goals of ‘Let’s Move’, the Healthy Weight Commitment partnership consisting of 16 of the leading food and beverage companies in the US, sold approximately 6.4 trillion fewer calories in 2012 than in 2007 [72]. An independent evaluation of the results, sponsored by the Robert Wood Johnson Foundation, concluded that total calories in the food supply were already declining by more than a trillion a year, and that the publicized “results” of the partnership would likely have been reached even in the absence of industry pledges.

Similarly, Australia’s Food and Health Dialogue was established as a public-private partnership to provide a framework for government, public health groups and industry to collaboratively “improve the availability of healthy food options and increase consumer awareness and understanding of the link between food choices and health outcomes” [73]. The Dialogue however has suffered from a lack of clear targets, independent evaluation and a plan for remediation if expectations are not met [74]. A recent study failed to identify any effects of the Dialogue on the knowledge, behaviours or nutrient intake of the Australian population or evidence of impact on diet-related disease [75].

### Public regulation of the private sector

The shortcomings of self-regulation and the inherent conflicts of interest of co-regulation lead many public health experts to categorically assert that public regulation is the only effective approach to achieve health-promoting change in industry practices [15]. This however presents the major challenge of enforceable public authority [76] over the private sector, and efforts to administer public sector control over the private sector in the interests of health promotion have met with resistance and resulted in mixed outcomes for public health. Such challenges are likely to increase with the impact of new trade and investment policies (free trade agreements) which have diminished the policy space within which Governments can exert control over NCD-related measures [77, 78].

Decades of public efforts to control tobacco consumption have revealed the extent of well-funded and well-orchestrated industry-led subversion to protect profitability [79]. As a result of litigation – the State of Minnesota versus Philip Morris (Supreme Court of Minnesota, 1996) –tobacco companies were required to open all previously secret records. The Tobacco Free Initiative revealed a decades-long campaign to subvert public policies through industry efforts to stop, slow, or delay the introduction of effective statutory tobacco control regulations. Similarly, the 1994 WTO adoption of the Trade-Related Aspects of Intellectual Property Rights – which dictates how states should regulate intellectual property protection – was the direct result of lobbying by powerful multinational corporations who succeeded in shaping international law to protect their markets [80]. Food, alcohol and beverage companies have at times adopted similar tactics (Table 1) – including in cooperation with each other. For example, analysis of tobacco industry documents has highlighted alliances between tobacco and alcohol industries in three areas – taxation, legal regulation and advertising/marketing restrictions [81].

**Table 1:** Corporate political activity [104]: how Industry Seeks to Influence Public Regulation, Public Evidence and Public Opinion

• Direct lobbying of decision-makers: Days before the publication of the 2003 WHO guidelines on healthy eating, which recommended that sugar should account for no more than 10% of a healthy diet, the Sugar Association wrote to the then WHO Director General, stating that it will “exercise every avenue available to expose the dubious nature” of the WHO’s report on diet and nutrition. The Association challenged WHO’s $406 m funding from the US and enlisted two US Senators to block the report [105].
• Using ostensibly independent front organizations, e.g. research institutes, trade associations: The American Dietetic Association (ADA), “devoted to improving the nation’s health” produces a series of Nutrition Fact Sheets – which industry sources pay for and take part in their writing [106]. ADA industry partners are provided access to key influencers and decision-makers, and outlets for research findings including professional meetings and scientific publications. In 2015, Coca-Cola was exposed as having provided significant technical and financial support to the nonprofit organization Global Energy Balance. The organization was criticized as little more than a front group – employing scientists to legitimize its message that obesity results from lack of exercise rather than poor diet [107].
• Strategic use of research, funding academics and public health bodies: An investigation by the British Medical Journal uncovered a “tangled web” of connections between the sugar industry and public health experts – through “research grants, consultancy fees and other forms of funding” [108]. A meta-analysis of available research showed clear relationships among the consumption of soft-drinks, poor nutrition and negative health outcomes. The meta-analysis demonstrated that studies funded by industry were more likely to find results favorable to industry [109] than studies funded from other sources. A review of corporate philanthropy by Coca-Cola and PepsiCo between 2011 and 2015 found they sponsored 95 public health organisations in the USA, including those dedicated to fighting diabetes, child health, and heart disease. During the same period, the two companies “lobbied against 29 public health bills intended to reduce soda consumption or improve nutrition” [110].
• Framing the debate: Industry consistently frames personal responsibility or lifestyle as the cause of unhealthy diet, and emphasizes physical activity and education as the most effective solutions. A variety of related messages are also typical of industry framing, including that companies offer choices and pleasure, emphasize moderation and do not encourage consumers to overuse their products [30].
• Discrediting opponents: The food industry often vilifies critics characterizing them as “food police”, leaders of a “nanny state”, and accuses them of desiring to strip people of their civil liberties [111].
• Using legal instruments to protect interests: the tobacco industry has regularly used the mechanism of bilateral investment treaties to “challenge states’ policymaking authority” [112] in controlling tobacco markets. For example, Philip Morris used legal channels to bring claims against the Governments of Australia and Uruguay, opposing their policies on tobacco labelling and plain packaging [78].

## Discussion

Over the last two decades, global health governance has undergone a striking transformation, characterized by a proliferation of global health institutions, the rise of emerging economies on the global stage, the escalation of transnational commercial activities as a determinant of poor health, and a marked turn in global health governance away from states and international organizations (IOs) towards private and hybrid public-private authority.

In this paper we posed three questions concerning the role of private authority in global health. Firstly, is the current architecture to govern commercial determinants of NCDs fit for purpose? We share a widespread and long-standing contention that in order to have the power and authority to address the commercial drivers of NCDs, the current global health architecture, particularly WHO and the World Health Assembly, needs reform, redesign and more resources [82]. There have been demands for the radical simplification of the health architecture [83], and for consideration of a single Multistakeholder Platform on Global Governance for Health, engaging governments, international organizations from a variety of sectors (including those driving health outcomes such as trade and investment, agriculture, environment, etc.), and non-state actors including civil society and business [26]. Chatham House proposed the creation of a UN-HEALTH modeled on UNAIDS, bringing together “all UN agencies working on global health issues” [84]. We support the establishment and/or strengthening of new governance platforms at the global as well as national levels, based on the collective power of multiple UN agencies, with clear rules of engagement in relation to conflicts of interest with the private sector, and an independent monitoring mechanism. Such a model likely has the best chance of promoting multisectoral, coherent [and powerful] action on the determinants of risk and illness – an approach that will be vital to achieving all health-related SDG targets, not just on NCDs [85].

The formation of new governance arrangements for controlling commercial determinants raises consideration of the second and third questions posed in this paper – the role that private authority should play, and the role of legal frameworks to protect the health of the public. We have reviewed the evidence around three models of regulating private authority in reaching public health goals, and highlighted deficiencies and challenges within each – these frequently include a lack of rigorous and systematic evaluation of public health impact for the models. We have not explored the fourth (unstated model) which is the absence of any kind of governance arrangement. Many have argued that for decades, this was the default position of the food industry, for example, which avoided any kind of regulatory activity beyond food hygiene/‘safety’ [86]. Our exploration of more active governance models has highlighted that neither self-regulation by industry nor statutory regulation alone are presently sufficient, and atomized public-private engagements or other forms of partnership are unlikely to provide a scalable model for tackling the transnational burden of NCDs. Nonetheless, in order to strengthen the three governance models, there are potential lessons to be learnt and applied from how the health sector has tackled other health issues, and legal and prescriptive frameworks already in existence that could be applied to the governance of commercial determinants of NCDs.

The UN has made a start on addressing the governance of commercial determinants of NCDs. With the 2011 UN High-Level Meeting on NCDs, NCDs became the second-ever health issue afforded this level of political attention at the General Assembly. A series of global NCD policy and coordination mechanisms have since been established, including a global monitoring framework [87], a global action plan, a UN interagency task force [88], and WHO’s global coordination mechanism - a multisectoral platform for engagement between public health bodies, civil society and the private sector [89]. This global level platform has yet to be evaluated, but at the national level a WHO survey of capacity for NCD prevention and control in 177 countries was published in 2015. Approximately half of countries reported a multisectoral policy in place to address NCDs and a third of countries reported having an “operational national multisectoral mechanism” [90]. However, the extent to which the private sector is included in these multisectoral mechanisms is not reported. Moreover, only a minority of countries (28 and 27% respectively) reported having policies in place to address food/beverage marketing to children or policies to limit trans-fats (with no such policies in place in low-income countries compared to 57% in high-income countries).

Whether any or all of these mechanisms will encourage meaningful national policy improvements, changes in industry practices and, ultimately, a reduction in NCDs, remains to be seen. The second UN High-Level Meeting on NCDs (2014) described progress as “insufficient” and “highly uneven” [91]. To encourage further progress, the 67th World Health Assembly established a working group to develop more detailed recommendations. In their report [92], the working group emphasizes the heterogeneity of the private sector, that conflicts of interest are not ubiquitous and often entities’ objectives may closely align with NCD prevention and control. Critically however, the working group states that effective government engagement with the private sector rests on strong regulatory frameworks, both statutory and self-regulatory, as well as a multistakeholder platform for implementation, monitoring and evaluation; a robust review mechanism; measures to encourage strong private sector contribution; transparent management of conflict of interest and; sharing of data to support collective action.

Given the inherent difficulties in public sector regulation operating alone, the preference of global health institutions for a “co-regulation” or pro-engagement approach may rest on several assumptions: that partnering with industry is the most effective way of aligning commercial incentives with consumer health interests; that mandatory regulation will jeopardise communication channels with business; and that regulatory options are not politically feasible [93]. Co-regulation also reflects historical reticence to subject corporations to supranational oversight and control. As Abbott and Snidal pointedly note, “[nation] states have denied virtually all [International Organizations] IOs direct access to private targets and strong regulatory authority” [94].

The UN system’s most significant achievement in controlling the commercial determinants of the NCD pandemic to date has been the 2003 Framework Convention on Tobacco Control (FCTC), one of the most widely embraced treaties in the history of the UN. The partial success of the FCTC in influencing national and global health policies has led to growing interest in new international legal instruments to address global health challenges. The complexity of NCDs however presents unique challenges to forging a comprehensive global response, such as those mobilized around tobacco or AIDS. The diversity of NCDs and their commercial drivers, including the multiplicity of industries involved, require multiple interventions, actors and policy responses across sectors, and are not easily reduced to legal principles. Similarly, the lack of a single ‘industry enemy’, the active role that food and beverage companies have taken to demonstrate their commitment to public health and the willingness of many public health actors to partner with these companies further complicates efforts to push for global regulatory frameworks.

The 2014 Report of the UN Special Rapporteur on the right to health noted “transnational corporations…have directly perpetrated serious human rights violations” [95]. The Special Rapporteur – an independent expert appointed by the Human Rights Council – explicitly called for the creation of an international mechanism to hold transnational corporations accountable for such abuses. The application of a human rights framework may offer a “logical, robust set of norms and standards… and add accountability mechanisms” for tackling NCDs [96] and has been widely applied to the realization of the right to health, including in some cases through the justiciability of economic and social rights as they have an impact on population health outcomes [97]. As such, accountability can be advanced through the use of existing UN human rights machinery such as the universal periodic reporting to the UN Human Rights Council and UN human rights treaty bodies. Notably, the UN Committee on Economic, Social and Cultural Rights has a long track record of issuing authoritative guidance on the right to health – which includes rights in relation to the determinants of health. Particular attention should be given to engaging the UN Special Rapporteur on the right to health to ensure that the office’s activity on NCDs is scaled up [98].

Specific national mechanisms for accountability are also warranted [99]. Beaglehole and colleagues [100] advocate national independent NCD accountability mechanisms, modeled on national AIDS commissions. Such mechanisms could provide valuable oversight to industry self-regulation and co-regulation initiatives to combat NCDs, monitor and evaluate national policies to regulate food, beverage and tobacco industries and their enforcement, and ensure remedial action.

Lessons could usefully be drawn from the experience of the Independent Expert Review Group (iERG) which reports to the UN Secretary-General on the results and resources related to the Global Strategy for Women’s and Children’s Health. The accountability framework has its origins in human rights functions—namely, monitoring, transparent and participatory review, remedy and action [101]. Others have proposed that national accountability mechanisms should include an emphasis on civic engagement [102] – and although the evidence for impact is still limited [103], the inclusion of the people and communities most affected is warranted on grounds of inclusivity and voice to create the political incentives for policy-makers to act.

## Conclusion

The ability of the global public health sector acting alone to influence the commercial determinants of NCD risk, or deal with NCD outcomes, is limited. Reaching SDG target 3.4 will necessitate interaction with, rather than exclusion of, the private sector, but such interaction needs to take into account not only the heterogeneity inherent in the ‘private sector’ (in terms of scale, nature of risk, and willingness to ameliorate public health impacts of those risks) but also the varied and potentially complex range of interactions and engagements that need to be addressed. While we, as public health professionals, believe that such interaction should be based on promoting evidence-informed interventions to meet globally agreed standards for public health goals, these are not the interests and objectives of all stakeholders involved in ‘engagement’ or ‘partnership’. The asymmetrical distribution of power and authority in these public-private interactions, and, hence, the overarching goals adopted, will depend, to a large degree, on the governance and accountability mechanisms put in place (and by whom). Our review of governance models for interaction between public and private sectors in relation to the commercial drivers of NCDs leads us to conclude that a sea-change in both governance and accountability is needed to prioritise and protect public health globally.

In Table 2 we summarise the criteria and conditions we think are necessary to safeguard the health of the public in relation to the commercial drivers of NCD risk. While the public health community may have strong views about the need for one type of governance mechanism for NCD risk over and above any other, the reality of the prevailing and powerful private sector interests are clear. Table 2 presents an agenda with a set of measures that the public health community should promote if we are to achieve public health targets and goals for all.

**Table 2:** Criteria, conditions and safeguards to govern commercial drivers of NCD risk: An agenda for the public health community

Self-regulation
Self-regulation will persist into the foreseeable future. With stronger consumer demand for healthier products, the tremendous leverage of industry could have a substantial impact on reducing population risk exposure. Private regulation can be anticipated particularly where states have weak oversight of transnational determinants of such risk. Nonetheless, as this paper has argued, the approach has proven to carry significant challenges to public health goals. Consequently, the public health community should advocate for, encourage and demand that such regulation exhibit the following four characteristics:
1) appropriate targets: ambitious targets/standards that are evidence-informed and rights-based, in the interest of public health, have been developed in a transparent manner (be they on product reformulation, promos, product placements, endorsements, marketing etc) and are SMART in nature (ie specific, measurable, attributable, realistic and time bound);
2) independent monitoring: review of compliance, progress and public health impact;
3) transparent reporting: with remedial action as necessary and an independent oversight body to ensure accountability;
4) sufficient scope for impact: include the leading corporate players and cover a significant proportion of the risk of exposure and the market—ideally applied globally to reduce cross border spillovers.
As such, self-regulatory regimes should have inputs from governments, scientists and civil society, particularly in target and standard-setting and ensuring accountability. Where these four conditions are not met, or where monitoring reveals low standards and lax enforcement, the public health community should seek to ensure that those risks are governed through hybrid or public regulation if at all possible.
Hybrid regulation
There is strong and widespread support for public-private partnership to address the overarching Agenda 2030 development framework and interest in leveraging such partnerships in health, including in the prevention of NCDs. We can only expect such cooperation to grow at country and global levels. But safeguards must be put in place to ensure public health concerns receive adequate attention. Consequently, the public health community should advocate for:
1) appropriate targets: ambitious targets/standard that are evidence-informed and rights-based, in the interest of public health and have been developed in a transparent manner (be they on product reformulation, promos, product placements, endorsements, marketing, etc) and are SMART in nature (ie specific, measurable, attributable, realistic and time bound);
2) independent monitoring: provide for an independent third party monitoring of compliance, progress and public health impact;
3) transparent reporting: with remedial action as necessary and an independent oversight body to ensure accountability;
4) sufficient scope for impact: include the leading corporate players (and should certainly not exclude firms were this to create an uneven and uncompetitive playing field) and cover a significant proportion of the risk of exposure and the market—ideally globally;
5) manage conflicts of interest: ensure that safeguards are in place to avoid potential or actual conflicts of interest or reputational threats to the public sector through partnership with firms or industries which do not conform to minimum acceptable standards (such as the principles set out by the United Nations Global Compact [113]); and
6) assess alternatives: ensure that the same objectives can’t be achieved more quickly and effectively through other means, and that the interests pursued by private partners would not threaten the longer-term public health objectives.
Public regulation
Public regulation is the favoured approach of many public health experts as they consider that experience with self- and hybrid-regulation has had insufficient public health impact on the prevention of NCDs. In the case of public regulation, the public health community should advocate for and advance the following conditions:
1) appropriate targets: ensure evidence-informed, rights-based targets for NCD risk mitigation that conform to international standards and laws, free of undue private sector influence—ones that stretch industry but are realistic and attainable and recognize that changes to industrial practices can take time;
2) inclusive target setting: ensure the mechanism to set and review targets and apportion roles and responsibilities including non-state actors and all relevant sectors of government so that regulation and action is focused on the fundamental drivers—it could be led by the Ministry of Health or through leadership in an overarching ministry such at the office of the Prime Minister, or the Ministry of Planning, etc.;
3) safeguards: ensure that measures and procedures are in place to manage apparent or real conflicts of interest and reputational risks and ensure a level playing field for non-state actors;
4) accountability: ensure independent monitoring, reporting and remedial action on progress as part of national efforts to report on implementation of Agenda 2030.

Ambitious NCD targets will remain nothing more than a chimera unless we adopt a new approach to governing commercial drivers which contribute to rising NCD levels globally. Building on the range of analyses and proposals already made, the international community must urgently envision and design a more robust architecture and support processes for accountability of NCD prevention. Moving forward, we need to ensure that a strengthened multistakeholder governance platform has the power to provide oversight of private authority, as well as engagement as appropriate. Accountability mechanisms for such a platform should be based on human rights principles and link to the relevant human rights machinery, with independent monitoring bodies reporting to a global platform and national NCD Commissions. We need a fundamentally new approach that can be discussed and adopted by world leaders at the 2018 UN High-Level Meeting on NCDs. This is a task that carries too much complexity, moral significance and political heft to leave to the WHA and WHO alone — as it involves nothing less than governing how we, and future generations, are able to live healthily.
